# Recurrent diphtheria outbreaks in Nigeria: A review of the underlying factors and remedies

**DOI:** 10.1002/iid3.1096

**Published:** 2023-11-28

**Authors:** Nasir Abdulrasheed, Lukman Lawal, Abdulazeez B. Mogaji, Ahmed O. Abdulkareem, Abdulrahman K. Shuaib, Sodiq G. Adeoti, Opeyemi P. Amosu, Abdulmujeeb O. Muhammad‐Olodo, Abdulwahab O. Lawal, Tawakalitu A. Jaji, Toufik Abdul‐Rahman

**Affiliations:** ^1^ Faculty of Clinical Sciences University of Ilorin Ilorin Nigeria; ^2^ MCON Research Institute Ilorin Nigeria; ^3^ Centre for Malaria and Other Tropical Diseases Ilorin Nigeria; ^4^ Toufik's World Medical Association Sumy Ukraine; ^5^ University of Ilorin Teaching Hospital Ilorin Nigeria; ^6^ Medical Institute Sumy State University Sumy Ukraine

**Keywords:** Africa, antitoxin, diphtheria−pertussis−tetanus, infectious disease, Nigeria, vaccine

## Abstract

**Introduction:**

The introduction of the diphtheria−tetanus−pertussis (DTP) vaccine into childhood immunization programs resulted in its widespread elimination in high‐income countries. However, Nigeria is currently experiencing an outbreak. The primary cause of diphtheria outbreaks and its high mortality rates in Nigeria was waning herd immunity due to low DTP coverage and a lack of diphtheria antitoxin (DAT), respectively. However, the underlying causes of Nigeria's low DTP coverage and DAT supply remain unknown.

**Method:**

Relevant studies and reports included in our review were obtained by a search through Google Scholar, PubMed, and organization websites using the terms “Diphtheria−Pertussis−Tetanus vaccine OR Diphtheria antitoxin and Nigeria OR Diphtheria Outbreak.” All articles considering diphtheria outbreaks, DTP vaccine, and DAT supply in Nigeria were considered without time restriction due to the paucity of data. We used the narrative synthesis approach to critically appraise, analyze, and draw inferences from the selected articles.

**Results:**

The main causes of low DTP coverage are insufficient supply, an inefficient cold chain system, and low uptake due to poor health literacy and negative sociocultural and religious beliefs, whereas the key barriers to DAT availability are insufficient production by pharmaceutical industries because of low demand and priority.

**Conclusion:**

The underlying causes of Nigeria's low DTP coverage and DAT supply are multifactorial. Both short‐term and long‐term measures are needed to control this outbreak and prevent future occurrences.

## Graphical abstract

- Main cause of Nigeria's diphtheria outbreaks is poor diphtheria vaccine coverage.
- Implicated factors are poor supply, inefficient cold chain system, and low acceptance.
- High mortality is attributable to nonavailability of antitoxin.
- Poor supply of antitoxin stems from low demand and high production costs.

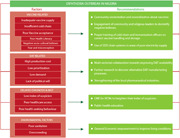

## 1 INTRODUCTION

Diphtheria is a highly infectious disease associated with grave morbidity and mortality.^1^ Infection with diphtheria is caused by strains of *Corynebacterium* species, mainly *Corynebacterium diphtheriae*, a nonmotile gram‐positive bacterium which is acquired via respiratory droplets and multiplies within the respiratory mucosa, releasing exotoxins that cause both local and systemic tissue injury.^2^

The global burden of diphtheria has long been markedly reduced in developed countries across the world, with significant control over the past decades in low‐ and middle‐income countries such as Nigeria.^3^ The introduction of the diphtheria vaccine into immunization programs has helped improve global efforts toward its eradication.^2^ However, Nigeria is currently experiencing an alarming surge in the number of recorded cases across the nation, a situation that calls for concern and indeed necessary intervention.^4^

According to the World Health Organization (WHO), mass vaccination is the primary control measure for diphtheria.^5^ The National Program on Immunization allows for a primary series of three doses of diphtheria−tetanus−pertussis (DTP) vaccine in infancy (at 6th, 10th, and 14th weeks), with booster doses after 10 years to cover for waning immunity.^6^ Studies by Ibrahim et al. and Besa et al. have shown that declining herd immunity due to low vaccine coverage is the key determinant of previous outbreaks in the country, while the high mortality rate recorded is attributable to the nonavailability of diphtheria antitoxin (DAT).^7^, ^8^

However, the underlying factors responsible for the low vaccine coverage in Nigeria and the barriers to the supply of DAT are yet to be examined. This review aims to critically appraise existing literature to highlight these problems and proffer practical and sustainable solutions. It also aims to highlight the importance of a higher index of suspicion in the early recognition and management of the disease.

## 2 METHODS

We conducted a comprehensive search for pertinent studies and reports in our review. We utilized Google Scholar, PubMed, and the websites of relevant organizations to find information using the search terms “Diphtheria−Pertussis−Tetanus vaccine OR Diphtheria antitoxin and Nigeria.” We included all articles that addressed diphtheria outbreaks, DTP vaccine, and DAT supply in Nigeria, without imposing any time limitations due to the limited availability of data. We used the narrative synthesis approach to critically appraise, analyze, and draw inferences from the selected articles.

## 3 EPIDEMIOLOGY OF DIPHTHERIA IN NIGERIA

Diphtheria was one of the deadliest diseases before the advent of vaccination, mainly affecting children under the age of 15, but it can also occur in adults over the age of 40. It had been essentially eliminated after the widespread introduction of the diphtheria toxoid vaccine in the mid‐20th century, especially in developed countries.^5^ Despite this, it remains a public health issue in developing countries, where there is poor coverage of the WHO Expanded Program on Immunization.^9^ India and other countries in Southeast Asia have contributed the bulk of the increasing incidence globally.^6^

The incidence of diphtheria is underreported in Africa. The number of cases reported in Nigeria has been on the decline: 3995 in 2000 and 1870 in 2018. There was a reported outbreak between February to November 2011 in Kimba and surrounding settlements in Borno State, north‐eastern Nigeria.^7^ There were 98 cases, the majority (63.4%) being <10 years of age. The case fatality ratio (CFR) was 21.4%, mostly among children aged 0−4 years (42.9%).^7^ This was followed by another outbreak reported from the Federal Medical Centre, Katsina State, Nigeria, during the COVID‐19 pandemic. Between July and December 2020, there were 35 cases (age range: 1.7−14 years). Twenty‐four deaths were recorded (CFR: 68.8%).^8^ The most recent outbreak started in May 2022 and is still ongoing. The Nigerian Centre for Disease Control (NCDC) confirmed it in December 2022. According to the NCDC's January situation report on diphtheria, there have been a total of 253 suspected cases in four states—north‐west (Kano), north‐east (Yobe), and south‐west (Lagos and Osun)—of the country, with 132 cases reported in January 2023 alone. Of these, 111 were confirmed cases, and the CFR is 19.8%.^10^

**Figure 1:**
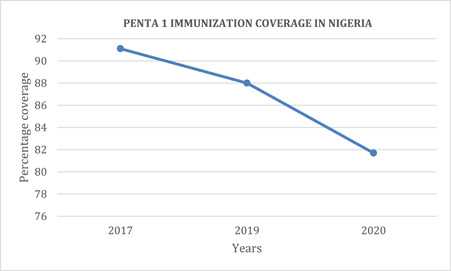
Graph showing the coverage of Pentavalent 1 vaccine in Nigeria. Data source: NHMIS (2023) (https://aho.afro.who.int/ind/af).

The principal method of preventing diphtheria in Nigeria is through vaccination. In Nigeria, the official National Immunization Schedule prescribes an initial series of three DTP vaccine doses during infancy (administered at the 6th, 10th, and 14th weeks of life). Subsequently, booster doses are recommended at 16−24 months, followed by another at 5−6 years of age, and then at 10 and 16 years to cover for declining immunity over time.^6^ The WHO advises that boosters should be customized to each country's disease epidemiology pattern.^5^ A poor vaccination coverage rate among the local population is a consistent factor in all these outbreaks in Nigeria. In the 2011 outbreak, there was <1% vaccine coverage of DTP among the study population.^7^ Likewise, the 2020 outbreak can be attributed to the same factor, although COVID‐19 contributed to the disruption of the vaccine supply chain at the time.^8^ The same pattern is evident in the current epidemic.^10^ Figure 1 depicts the pentavalent‐1 immunization coverage rate between 2017 and 2020 in Nigeria, dropping below the WHO's recommended 90%.^5^ The high CFR seen in some of these outbreaks can be attributed to the scarcity of DAT, as Nigeria does not stock DAT.^6^, ^8^

## 4 OVERVIEW OF CLINICAL CHARACTERISTICS AND MANAGEMENT OF DIPHTHERIA

The bacteria act by toxin production, which inhibits cellular protein synthesis and is thought to cause local tissue destruction and formation of the characteristic adherent pseudomembrane at the affected sites. *C. Diphtheriae* has an average incubation period of 2−5 days but can be up to 10 days. It can affect any mucous membrane.^11^

Two forms have been described in the literature, viz: the respiratory and non‐respiratory forms, with the former carrying a higher risk of mortality. The most common type of diphtheria is the respiratory form, classified clinically based on the parts affected as; pharyngeal, tonsillar, laryngeal, and nasal diphtheria.^11^ It is characterized by prodromal symptoms of low‐grade fever (usually less than 38.3°C), runny nose, sore throat, conjunctivitis, cough, malaise, and anorexia. When severe, the pseudomembrane forms, usually on the tonsils and extends locally to surrounding areas. This extensive membrane formation can cause airway obstruction, hoarseness of voice, barking cough, and a marked submandibular edema and lymphadenopathy that gives a characteristic “bull neck” appearance. According to Ibrahim et al., the presence of a “bull neck” appearance was reported to be a positive predictor of death in the 2020 outbreak in Nigeria. This may suggest a need for close monitoring of patients with such features.

There is also the non‐respiratory type of diphtheria, which includes the cutaneous form that is characterized by scaly rash and ulcers with well‐demarcated edges that sometimes exist along the lines of chronic skin lesions, and other rare forms that involve mucous membranes of the conjunctiva, auditory canal, and vulvovaginal region. These types are mostly caused by nontoxigenic *Corynebacterium* species. The commonest complications of the infection are myocarditis and polyneuropathies. Other complications are nephritis, corneal scarring (worsened by vitamin A deficiency), encephalitis, diarrhea, pneumonia, and subacute sclerosing panencephalitis.^7^, ^8^, ^11^

The NCDC guidelines define a suspected case of diphtheria as any person with an illness of the upper respiratory tract characterized by the following; pharyngitis, nasopharyngitis, tonsillitis, or laryngitis AND adherent pseudomembrane of the pharynx, tonsils, larynx, and/or nose, while a laboratory‐confirmed case is a person with *Corynebacterium* spp. isolated by culture and positive for toxin production (the modified Elek test), regardless of symptoms.^12^

The disease is managed by administering DAT and antibiotic therapy (penicillin or erythromycin); however, it can be fatal in 5%−10% of cases. The case fatality is reported to be higher in settings where DAT is unavailable. Regrettably, this is the situation in Nigeria, where the case fatality rate was 68.8% in 2020 and about 20% in the ongoing outbreak.^7^, ^12^ The extent of the outbreaks and their severity calls for an urgent investigation of the underlying causes to inform the prompt implementation of effective control measures.

## 5 DIPHTHERIA OUTBREAK; WHAT IS NIGERIA NOT DOING RIGHT?

The fact that there is an outbreak of a vaccine‐preventable disease underscores a fundamental problem with the coverage of childhood immunization in Nigeria. Unvaccinated children are highly vulnerable to vaccine‐preventable diseases, and this is especially true for Nigeria, where about 14% of the global burden of children with zero‐ or missed‐dose of vaccines reside.^13^ The peculiar challenges driving the current outbreak of diphtheria in Nigeria are multilayered, and they include:

### 5.1 Problems related to vaccination

Primarily, there are barriers to vaccination; in certain parts of the country, especially the Northern region, there are some cultural and religious beliefs against taking vaccines, with resultant low acceptance and uptake of immunization, including diphtheria‐toxoid‐containing pentavalent vaccine.^8^ Furthermore, health behaviors are influenced by an array of factors at the individual, household, community, and societal levels.^13^ Factors like the child's place of birth, age, birth order, ownership of an immunization card, mother's age, level of education, religion, conjugal power as well as community perception of immunization all influence attitude to vaccination.^13^ More so, poor health literacy was found to influence attitudes to vaccines, intention to get vaccinated, and vaccine uptake. In the northern part of the country where these variables tend to be negative, vaccination is at an all‐time low, with resultant bursts of epidemics like the current diphtheria outbreak. Also, fear and misconception about vaccination are other factors that affect uptake.^13^ These predispose children and young people in this region to infection by *C. diphtheriae*. This may also explain why about 95% of the reported cases and 92% of the deaths in this current outbreak are in the North (Kano and Yobe).^7^, ^14^, ^15^

Additionally, inadequate vaccination coverage; nonavailability of diphtheria‐toxoid‐containing pentavalent vaccines for either the first or booster doses resulting in lack of, or poor childhood vaccination is a major factor responsible for this outbreak.^7^, ^8^, ^13^, ^15^, ^16^, ^17^ Even when vaccines are available, the perennial subpar cold chain system in the country means vaccines cannot be adequately preserved, evidenced most recently by the failure of the country to meet the requirement to stock up and vaccinate its citizens against COVID‐19.^18^ There is equally prevalent illiteracy among the most affected population, and this is especially true for the girl child. It is therefore not surprising that there is low vaccination coverage in the region, reflecting the low maternal education and literacy.^13^ It is estimated that over 50% of Nigerian parents are ignorant of vaccination schedules.^19^ More so, some remote and hard‐to‐reach communities are not covered because of poor accessibility.^8^ In the current outbreak, only 10.8% of the affected children are vaccinated against diphtheria.

### 5.2 Delayed diagnosis and management

One of the major issues is the delayed presentation to health facilities. Factors contributing to this include endemic poverty and a low standard of living among the affected people, which reflect the poor economic status of the country. According to Statista, nine of the top 10 states with high rates of poverty in Nigeria are in the northern region.^20^ It is therefore not surprising that diphtheria outbreaks are also more prevalent (97.6%) in this region. These contribute to poor health‐seeking behavior and therefore, late presentation to health facilities. In addition, poor knowledge of the symptoms and signs of the disease among the affected population is also reported to cause delayed presentation, increased spread, and higher fatality.^8^

Another critical factor is a low index of suspicion among health workers. It often takes some time before the first case in any outbreak is picked up, mostly because clinicians do not expect to see such a case as is often uncommon.^8^, ^12^ The preceding decline in the incidence of diphtheria over the years may have led to a decrease in its index of suspicion among clinicians, which has been associated with the under‐reporting of the disease in developing countries. In the 2011 outbreak in Nigeria, none of the 14 cases treated at a general hospital was diagnosed as diphtheria; each received a working diagnosis of mumps or parotitis.^7^ More so, none received the appropriate antibiotics or DAT. This delay is associated with the potential for the spread of the disease, resulting in an outbreak.^7^

Furthermore, most of the outbreaks occur in places where there is poor access to healthcare, inadequate personal protective equipment (PPE) for healthcare workers (HCWs), and/or lack of laboratory capacity to make a diagnosis.^8^, ^13^ Even when the diagnosis is made on clinical grounds, there is usually no adequate treatment in the form of DAT and appropriate antibiotics.^8^

### 5.3 Barriers to DAT supply

Studies in Nigeria have found that antitoxin's unavailability was a major cause of diphtheria‐related mortality in the study population.^21^ Historically, the pharmaceutical industry has struggled to consistently produce drugs that are not in high demand. Pharmaceutical companies' reduced production of DAT could be attributed to the effective incorporation of control and preventive measures in advanced countries, resulting in a decreased commitment to fighting what does not appear to be a threat in their respective locales. This rising trend of inadequacy puts additional strain on dependent countries with struggling healthcare systems, such as Nigeria.

Furthermore, more emphasis is placed on the prevention of diphtheria through vaccination than its management, which may have further affected the DAT chain of supply. In Nigeria, for instance, the stock of DAT is not a major concern considering the high vaccine effectiveness. This may have diminished the pharmaceutical industry's interest in large‐scale DAT production. Moreover, the WHO identified stringent regulatory requirements in managing blood‐derived products as a major concern that has hampered the production and availability of DAT. In the first half of the 20th century, clonal techniques were used to produce DAT for national use, but routine production was slowed due to ethical concerns and widespread vaccine coverage.^22^, ^23^

### 5.4 Environmental factors

Environmental factors play a significant role in the occurrence and transmission of diphtheria in Nigeria. The country's diverse ecological zones and varying climate patterns directly impact the disease's prevalence. Inadequate sanitation and access to clean water in certain regions create ideal breeding grounds for the diphtheria‐causing bacterium, *C. diphtheriae*. Overcrowded living conditions in urban areas exacerbate the problem, facilitating the rapid spread of the disease. Additionally, environmental pollution and air quality issues can weaken individuals' respiratory systems, making them more susceptible to respiratory infections like diphtheria.^13^, ^14^ Therefore, efforts to combat diphtheria in Nigeria must include improvements in sanitation, hygiene education, access to clean water, and addressing environmental pollution to reduce the disease's impact on public health.

### 5.5 Lack of political will

The recurrent diphtheria outbreaks in Nigeria can be attributed in part to the lack of political will to implement and sustain effective public health measures. Diphtheria, a preventable disease through vaccination, persists due to inadequate funding, weak healthcare infrastructure, and insufficient commitment from authorities.

Furthermore, corruption and mismanagement in the healthcare system exacerbate the problem, diverting resources away from diphtheria prevention efforts. Without a strong political commitment to prioritize vaccination programs, strengthen healthcare infrastructure, provide adequate supply of DAT, and combat corruption, Nigeria continues to grapple with recurrent diphtheria outbreaks, endangering the lives of its citizens.^19^

## 6 IMPLICATIONS OF DIPHTHERIA OUTBREAK IN NIGERIA

The diphtheria outbreak could have catastrophic consequences if left unchecked.^24^ The public's initial reaction to the epidemic is one of fear, uncertainty, and widespread misinformation. A lack of appropriate information contributes to stigma, delayed presentation to healthcare facilities, a worsening prognosis for affected individuals, and an increase in the likelihood of further spread.^8^ If this outbreak is not contained, the propensity of the disease to thrive in unimmunized school‐age children will significantly increase under‐5 mortalities in Nigeria.^21^

Moreover, the rising incidence of diphtheria further stresses the already strained nation's healthcare system. The high cost of financial and human resources required to obtain the DAT and implement preventive interventions would have an impact on the country's economy, exacerbating the severity of poverty among the masses.^24^ Furthermore, uncontrolled infection spread, both intra‐national and cross‐border, could stymie trade and negatively impact the country's economy.

HCWs are highly susceptible to contracting the disease as they are the first line of contact with patients. A sick HCWs contributes to less manpower, reducing the capacity of the healthcare system to adequately respond to emergencies. Also, an HCW may be a carrier, harboring the pathogen and unknowingly spreading the infection, thereby increasing the disease burden. All these underscore the need for urgent coordinated action to mitigate these problems.

## 7 THE WAY FORWARD

Given that diphtheria is a vaccine‐preventable disease, this outbreak necessitates immediate action in terms of vaccine supply, cold chain system management, levelling barriers to vaccination, and ensuring DAT availability.

**Figure 2:**
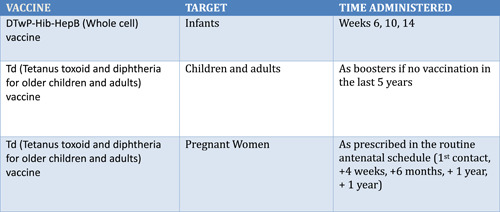
Current vaccination schedule for young children, adolescents, and adults in Nigeria. WHO (2023) Source: http://immunizationdata.who.int/pages/schedule-by-country/nga.html?DISEASECODE=DIPHTHERIA+PERTUSSIS+TETANUS&TARGETPOP_GENERAL=

Figure 2 provide information on the current immunization schedule across all ages and vulnerable population in Nigeria. Obstacles against adequate coverage of this vaccination schedule must be addressed. Cultural and religious leaders have a profound influence on people's perceptions of vaccination. In Nigeria religious and traditional leaders are custodians of faith and culture, respectively and wield great influence on devotees, making them veritable tools for changing people's attitudes. These institutions can be deployed to boost the vaccination drive.^25^ Thus, they should be incorporated as part of the community team to ensure widespread coverage and acceptance in our environment. During outbreaks, timely and widespread immunization efforts, prioritizing affected areas and high‐risk populations, are instrumental in advancing vaccine coverage, curtailing diphtheria outbreaks, and protecting vulnerable individuals, especially children, from severe illness and potential fatalities. Fears and misconceptions could be allayed by deploying a multipurpose framework aimed at individuals, sociopolitical situations, and specific social groupings. Effective strategies for increasing public trust and confidence in vaccines include good policies, transparency, accountability, diplomacy, and public participation in vaccine clinical trials, approval, and purchase. Proper health literacy advocacy initiatives must also be used as a tool to raise public awareness of diphtheria outbreaks by providing accurate facts and details, as well as educating the public on vaccine safety and effectiveness. It is also crucial to employ a socially sensitive vaccination promotion strategy, involving social media influencers in vaccination campaigns and disseminating accurate information.^26^

Second, the Nigerian government should collaborate with pharmaceutical companies, global leaders, and other relevant stakeholders in a concerted effort to ensure widespread availability and easy access to diphtheria vaccines, as well as make funds available to ensure manufacturing capacity expansion. The government should also partner with and support community organizations to conduct extensive and well‐mannered community engagement.

Third, an efficient cold chain system is paramount to the success of any vaccination program. The challenge of poor electricity supply needed for cold storage and distribution can be overcome by using solar direct‐drive refrigerators (SDD). This SDD technology directly utilizes solar energy, cold storage materials, and energy stored in the frozen bank to keep the refrigerator cold during the night and on cloudy days.^18^ As a result, the continent's geographic location, which lies within the tropical belt, is capitalized to promote vaccine equity. This solar technology's cost‐effectiveness and sustainability make it a promising solution to the electricity challenge. Such objectives must be strongly supported by the Global Alliance Vaccine Initiative, the WHO, and other multinational public health organizations promoting health equity in low‐ and middle‐income countries.^18^ Additionally, vaccine cold‐chain and immunization officers must be given proper training in vaccine handling and storage. They must also be educated on correct data management and Geographic Information System maps, which can provide information about hinterlands and key structures, such as religious centers, recreational centers, and markets, all of which are target groups for mass outreach campaigns. These would help to manage logistical issues with vaccine distribution and assist in efficient microplanning for the vaccination process.

Fourth, the NCDC should raise awareness among HCWs regarding the importance of maintaining a high level of suspicion for diphtheria. This will help prevent unnecessary delays in diagnosing and treating the disease. Given that numerous misdiagnoses with mumps were noted during one of the previous outbreaks, the importance of throat examination for the classic pseudomembrane should be stressed for any suspected cases. It's crucial for HCWs to promptly isolate suspected cases and inform the local government's Disease Surveillance and Notification Officer, who will then alert the State Ministry of Health, ultimately escalating the situation to the NCDC. Additionally, it's essential to track and quarantine close contacts of confirmed cases. To protect HCWs during diphtheria outbreaks, prioritize their safety through diphtheria vaccination and enforce strict infection control measures, including appropriate use of PPE, patient isolation, and rigorous hygiene practices. Ongoing training and awareness campaigns can further enhance HCWs safety when dealing with diphtheria cases.

Fifth, relevant stakeholders are hereby encouraged to renew their positions on DAT production and supply. Adequate investment in further research to develop alternative methods of antitoxin production might reduce the ethical demands and the cost of producing antitoxins from blood‐derived products. The Nigerian government should establish a functional collaboration with other affected countries, particularly those in Africa, by forming a strong alliance with pools of financial resources and funding strategies that will provide a reasonable avenue to seek antitoxin production from pharmaceutical companies under agreeable terms and conditions. In addition, African leaders should partner with the WHO, global leaders, policymakers, and multinational pharmaceutical companies to strengthen the capacity of their local pharmaceutical industries to produce sufficient doses of DAT through the development of research and technical know‐how, technology sharing, patent waivers, and investments. The presence of DAT plants in Nigeria will aid in adequate and dependable response to outbreaks due to improved availability and proximity.

Lastly, socioeconomic development is sine qua non to the success of health equity. With low socioeconomic status as evidenced by poverty, overcrowding, and poor hygiene, the diphtheria pathogen thrives. Prior studies suggest that vaccination percentages may be reduced among demographically defined groups of lower education and income levels.^27^ The Government should make living conditions favorable by providing basic amenities, education, and formulating relevant policies, laws, and regulations.

## 8 CONCLUSION

Diphtheria is a fatal, vaccine‐preventable disease caused by strains of *C. diphtheriae*. Nigeria is currently witnessing an outbreak of the disease with serious morbidity and mortality. Factors found to contribute largely to this outbreak include the unavailability of the DPT vaccine, inefficient cold chain system, cultural and social beliefs militating against taking vaccines, delayed diagnosis and management, a low index of suspicion among health workers, inadequate DAT production, poor living conditions, and a lack of political will.

Short‐ and long‐term measures are required to control this outbreak and prevent future occurrences. There is a need for concerted and coordinated efforts from the local to the regional and global levels aimed at increasing DTP vaccine availability and demystifying religious and cultural beliefs against vaccination while collaborating with key stakeholders to ensure DAT's easy availability and accessibility in Nigeria and reducing Nigeria's over‐dependence on developed nations for emergency aid. The government should also ensure the provision of basic amenities and work to improve the country's socioeconomic status.

## AUTHOR CONTRIBUTIONS

Lukman Lawal and Nasir Abdulrasheed conceptualized and designed the study. All authors performed data acquisition, analysis, and interpretation. All authors drafted the article. Lukman Lawal, Nasir Abdulrasheed, Abdulazeez B. Mogaji, and Toufik Abdul‐Rahman critically reviewed the article for important intellectual content. All authors approved the final draft of the article for submission. Lukman Lawal and Nasir Abdulrasheed should be considered as joint first author.

## CONFLICT OF INTEREST STATEMENT

The authors declare no conflict of interest.

## Data Availability

No new data was generated.

## References

[1] Sharma NC , Efstratiou A , Mokrousov I , Mutreja A , Das B , Ramamurthy T . Diphtheria. Nat Rev Dis Primers. 2019;5:81. 10.1038/s41572-019-0131-y 31804499

[2] World Health Organization . Diphtheria: vaccine preventable diseases surveillance standards. 2018. Accessed February 7, 2023. https://www.who.int/publications/m/item/vaccine-preventable-diseases-surveillance-standards-diphtheria

[3] Clarke KEN , MacNeil A , Hadler S , Scott C , Tiwari TSP , Cherian T . Global epidemiology of diphtheria, 2000–2017. Emerging Infect Dis. 2019;25:1834‐1842. 10.3201/EID2510.190271 PMC6759252 31538559

[4] Nigeria Centre for Disease Control and Prevention . Diphtheria health advisory for health care workers amidst outbreak in Nigeria. 2023. Accessed February 7, 2023. https://ncdc.gov.ng/news/436/diphtheria-health-advisory-for-health-care-workers-amidst-outbreak-in-nigeria

[5] Clarke KE . Review of the Epidemiology of Diphtheria 2000‐2016. World Health Organization; 2017:2‐4. https://cdn.who.int/media/docs/default-source/immunization/sage/2017/sage-meeting-of-april-2017/background-docs/session-diphtheria/1.-review-of-the-epidemiology-of-diphtheria---2000-2016-pdf-829kb.pdf?sfvrsn=9ba4f061_3

[6] Abubakar MY , Lawal J , Dadi H , Grema US . Diphtheria: a re‐emerging public health challenge. Int J Otorhinolaryngol Head Neck Surg. 2019;6:191‐193. 10.18203/ISSN.2454-5929.IJOHNS20195713

[7] Besa NC , Coldiron ME , Bakri A , et al. Diphtheria outbreak with high mortality in northeastern Nigeria. Epidemiol Infect. 2014;142:797‐802. 10.1017/S0950268813001696 23866913 PMC3942815

[8] Ibrahim O , Lawal I , Mohammed B , et al. Diphtheria outbreak during Covid‐19 pandemic in Katsina, North‐Western Nigeria: epidemiological characteristics and predictors of death. Nigerian J Basic Clin Sci. 2022;19:59. 10.4103/NJBCS.NJBCS_35_21

[9] Rintani A , Mintarsih T , Muliawan YMRB , Siregar JS , Widodo AP . Risk factors associated to diphtheria outbreak in developing countries. Jurnal Ilmu Kesehatan Masyarakat. 2018;9:83‐95. 10.26553/JIKM.2018.9.2.83-95

[10] Nigeria Centre for Disease Control and Prevention . January 2023 update on diphtheria. 2023. Accessed March 10, 2023. https://ncdc.gov.ng/diseases/sitreps

[11] Acosta AM , Moro PL , Susan HP , Tiwari TSP . Diphtheria. The Epidemiology and Prevention of Vaccine‐Preventable Diseases. Centers for Disease Control and Prevention; 2021:97‐110. https://www.cdc.gov/vaccines/pubs/pinkbook/dip.html

[12] Nigeria Centre for Disease Control and Prevention . Diphtheria. 2023. Accessed February 4, 2023. https://ncdc.gov.ng/diseases/info/D

[13] Mahachi K , Kessels J , Boateng K , et al. Zero‐ or missed‐dose children in Nigeria: contributing factors and interventions to overcome immunization service delivery challenges. Vaccine. 2022;40:5433‐5444. 10.1016/J.VACCINE.2022.07.058 35973864 PMC9485449

[14] The Guardian Nigeria News . NCDC confirms 123 diphtheria cases, 38 deaths in 4 states. 2023. Accessed February 4, 2023. https://guardian.ng/news/ncdc-confirms-123-diphtheria-cases-38-deaths-in-4-states/

[15] Osaji S. Lack of childhood immunization fuelling diphtheria—NCDC 2023. 2023. Accessed February 4, 2023. https://punchng.com/lack-of-childhood-immunisation-fuelling-diphtheria-ncdc/

[16] Abujah R. Nigeria records 253 cases of suspected diphtheria in 2 months—NCDC 2023. 2023. Accessed February 4, 2023. https://sciencenigeria.com/nigeria-records-253-cases-of-suspected-diphtheria-in-2-months-ncdc/

[17] Joel M. FACTSHEET: what you should know about the diphtheria outbreak in Nigeria‐Africa check 2023. 2023. Accessed February 4, 2023. https://africacheck.org/fact-checks/factsheets/factsheet-what-you-should-know-about-diphtheria-outbreak-nigeria

[18] Lawal L , Aminu BM , Murwira T , et al. Low coverage of COVID‐19 vaccines in Africa: current evidence and the way forward. 2022;18. 10.1080/21645515.2022.2034457 PMC9009957 35240908

[19] Oleribe O , Kumar V , Awosika‐Olumo A , Taylor SD . Individual and socioeconomic factors associated with childhood immunization coverage in Nigeria. Pan African Med J. 2017;26:220. 10.11604/PAMJ.2017.26.220.11453 PMC5491752 28690734

[20] Sasu DD. Nigeria: poverty rate, by state. Statista 2022. 2022. Accessed February 4, 2023. https://www.statista.com/statistics/1121438/poverty-headcount-rate-in-nigeria-by-state/

[21] Sadoh AE , Sadoh WE . Diphtheria mortality in Nigeria: the need to stock diphtheria antitoxin. African J Clin Exp Microbiol. 2011;12. 10.4314/ajcem.v12i2.64323

[22] United Nations Children Fund . Diphtheria antitoxin: market update. 2017. Accessed March 4, 2023. https://www.unicef.org/supply/sites/unicef.org.supply/files/2019-06/diphtheria-antitoxin-market-update.pdf

[23] World Health Organization . Diphtheria anti‐toxin (DAT) supply issues: brief review and proposition. 2017. Accessed March 4, 2023. https://cdn.who.int/media/docs/default-source/immunization/sage/2017/sage-meeting-of-april-2017/background-docs/session-diphtheria/3.-supply-of-diphtheria-antitoxin-pdf-204kb.pdf?sfvrsn=2bd062e3_3

[24] Madhav N , Oppenheim B , Gallivan M , Mulembakani P , Rubin E , Wolfe N . Pandemics: risks, impacts, and mitigation. Improving Health Reducing Poverty. 2017;9:315‐345. 10.1596/978-1-4648-0527-1_CH17 30212163

[25] Adebiyi K . Role of traditional, religious leaders in upscaling COVID‐19 vaccination—EnviroNews Nigeria 2021. 2021. Accessed February 9, 2023. https://www.environewsnigeria.com/role-of-traditional-religious-leaders-in-upscaling-covid-19-vaccination/

[26] World Health Organization . Solar direct‐drive vaccine refrigerators and freezers. 2017. Accessed February 10, 2023. https://www.who.int/publications/i/item/WHO-IVB-17.01

[27] Lazarus JV , Ratzan SC , Palayew A , et al. A global survey of potential acceptance of a COVID‐19 vaccine. Nature Med. 2020;27:225‐228. 10.1038/s41591-020-1124-9 33082575 PMC7573523

